# Laparoscopic *versus* open repair of perforated peptic ulcer: systematic scoping review and in-depth evaluation of existing evidence

**DOI:** 10.1093/bjsopen/zrae163

**Published:** 2025-03-06

**Authors:** Katy A Chalmers, Matthew J Lee, Sian E Cousins, Adam Peckham Cooper, Peter O Coe, Natalie S Blencowe

**Affiliations:** Bristol Centre for Surgical Research, University of Bristol, Bristol, UK; Institute for Applied Health Research, College of Medical and Dental Sciences, University of Birmingham, Birmingham, UK; Department of Trauma and Emergency General Surgery, University Hospitals Birmingham NHS Foundation Trust, Birmingham, UK; Bristol Centre for Surgical Research, University of Bristol, Bristol, UK; Leeds Institute of Emergency General Surgery, Leeds Teaching Hospital NHS Trust, Leeds, UK; Department of Upper Gastrointestinal Surgery, Leeds Teaching Hospital NHS Trust, Leeds, UK; Bristol Centre for Surgical Research, University of Bristol, Bristol, UK; Leeds Institute of Emergency General Surgery, Leeds Teaching Hospital NHS Trust, Leeds, UK

## Abstract

**Background:**

Perforated peptic ulcer remains a common contributor to morbidity and mortality rates worldwide. In common with other emergency surgery conditions, there is a trend towards minimally invasive surgery. This review aims to describe current evidence comparing open and laparoscopic management strategies for perforated peptic ulcers, by summarizing patients, intervention, comparator, outcomes, describing intervention components and delivery, outcomes reported and assessing study pragmatism (applicability) using PRagmatic Explanatory Continuum Indicator Summary-2.

**Methods:**

Systematic searches of published literature were performed using Ovid MEDLINE and Embase online databases, as well as clinical trial databases. Randomized trials comparing laparoscopic and open repair of peptic ulcer were included. Data extracted included study metadata, patients, intervention, comparator, outcomes elements, technical aspects of interventions and use of co-interventions, and surgeon learning curves/experience. Applicability was assessed using the PRagmatic Explanatory Continuum Indicator Summary-2 tool, to explore whether trials were predominantly pragmatic or explanatory, and study validity assessed using the Cochrane Risk-of-Bias 2 tool.

**Results:**

A total of 408 studies were screened for eligibility, with nine finally included (880 patients). Incision, ulcer closure details and lavage were the most frequently reported aspects of laparoscopic repair. Co-interventions such as antibiotic use and analgesia were reported in most articles, whilst nutrition and *Helicobacter pylori* eradication were not reported. Interventions were generally delivered by high-volume laparoscopic surgeons. Studies were considered at high Risk-of-Bias. PRagmatic Explanatory Continuum Indicator Summary-2 assessment found studies were neither fully pragmatic nor explanatory.

**Conclusion:**

Laparoscopic repair of perforated peptic ulcer is a variably defined intervention. Consideration of how intervention components and co-interventions should be optimally delivered is required to facilitate a well designed randomized trial.

## Teaser

An in-depth examination of existing evidence comparing laparoscopic and open repair of perforated peptic ulcer demonstrates that current evidence is based on small, single-centre randomized clinical trials, using heterogenous interventions and comparators, poorly described co-interventions, and outcomes of limited patient importance. Consideration of how intervention components and co-interventions should be optimally delivered is required to facilitate a well-designed trial.

## Introduction

Perforated peptic ulcer (PPU) is an important condition, accounting for the highest mortality rates for emergency surgery patients in the Global Burden of Disease study^1^. In the UK, PPU repair was the sixth most common surgical procedure (approximately 1000 patients) performed during emergency laparotomy^2^ and conferred a 30-day mortality rate of 10.6%^2^. The first laparoscopic repair of PPU was performed in 1989^3^, and since then proponents have suggested theoretical benefits over the open approach. Such advantages include smaller incisions, reduced postoperative pain, reduced intra-abdominal manipulation (minimizing postoperative ileus), as well as earlier patient mobilization, hospital discharge and return to usual activities. Laparoscopic surgery is also thought to decrease risks of future problems with adhesions^4^. Conversely, however, there is some evidence to suggest that the laparoscopic approach may confer other problems such as limited lavage, recurrent leaks from the repair and a longer procedural time^5,6^. Furthermore, necessary skills for laparoscopic repair may be limited across general surgeons.

Systematic reviews comparing laparoscopic and open PPU repair have previously been conducted^5,6^. These found favourable outcomes associated with laparoscopic repair across domains such as morbidity rate and duration of stay, with no difference in leak rates between laparoscopic and open repair^7^. Studies on real-world data have had similar findings^6,8^. Whilst clinical outcomes are documented, the metholodogical quality is unclear and details about the interventions under evaluation (for example how a patch is secured, port sites, adjunct treatments) and the expertise of clinicians performing the operation were not examined. This limits the applicability and transferability of these studies into clinical practice.

Given the current limitations of the synthesized evidence for approaches to PPU repair, an up-to-date comprehensive review is needed, which examines the methodological quality and applicability of the randomized clinical trials (RCTs) using appropriate and contemporary tools. This review aims to describe randomized evidence comparing open and laparoscopic management strategies for PPUs, by summarizing patients, intervention, comparator and outcomes (PICO), describing intervention components and delivery, outcomes used and assessing study applicability using PRagmatic Explanatory Continuum Indicator Summary (PRECIS-2).

## Methods

A systematic review was performed. The study was reported according to the framework set out by PRISMA^9^ and was registered with an international prospective register of systematic reviews (PROSPERO) (CRD42023404537). A protocol was not published.

### Study eligibility

Included studies were RCTs comparing laparoscopic and open approaches for adult (≥18 years) patients with a preoperative diagnosis of PPU (gastric or duodenal). Studies using any mechanical ulcer repair method (for example patch, primary suture) were included. Conference abstracts were excluded due to the high chance of missing information.

### Systematic searches

Systematic searches of Ovid MEDLINE and Embase were performed between 1 January 1990 and 15 December 2022. A comprehensive search strategy was developed using concepts for ‘laparoscopic’, ‘open’, ‘surgery’, ‘peptic ulcer’ and ‘randomised clinical trial’ (*Table S1*). Titles, abstracts and full-text manuscripts were screened by two of three independent reviewers (N.S.B., K.A.C., S.E.C.), with disagreements considered through re-discussion and involvement of the third reviewer. Systematic searches of clinical trial registry databases (clinicaltrials.gov, International Clinical Trials Registry Platform (ICTRP) and International Standard Randomised Controlled Trial Number (ISRCTN)) were performed to identify any eligible ongoing studies that had completed but not yet published. Search terms were broad to minimize the chance of missing relevant trials. Trial entries were screened by two independent reviewers (K.A.C., S.E.C.) with disagreements resolved by a third reviewer.

### Data collection

Data were extracted in the following categories:

#### Key study characteristics

The year of publication and country in which the study was undertaken were extracted, as well as the total number of participants randomized and the number of centres.

#### Details of the patient PICO

The inclusion/exclusion criteria were extracted for each study. The name of the intervention and comparator were extracted and examined in more detail (described below). Primary and secondary outcomes were recorded. Where no primary outcome was specified, the first outcome reported in the results section of the abstract was assumed to be the primary outcome. It was also recorded which outcomes were reported in both methods and results sections of the article, methods only or results only.

#### Details of intervention components and co-interventions

Technical descriptions of the component parts of both intervention and comparator were extracted by four independent reviewers with expert clinical knowledge (N.S.B., P.O.C., M.J.L., A.P.C.). Initially, reviewers drew on their own experiences with performing the open and laparoscopic procedures to identify the components of the interventions, including co-interventions. These were then discussed to identify omissions and clarify disagreements. A preliminary framework for intervention/comparator components and co-interventions was prepared by the reviewers, based on an existing typology^10^. Intervention components included access to the abdomen, confirmation of diagnosis, assessment of ulcer, ulcer closure method, management of contamination and abdominal wall/superficial closure. Co-interventions included patient/surgeon positioning, antibiotics, analgesia and introduction of fluids.

Descriptions of each component, if reported, were then extracted independently in duplicate for each identified study. Disagreements were resolved through re-discussion and involvement of a third reviewer if required. Any additional components identified during this process were added to the framework and applied to all included papers until no further changes were necessary.

#### Details of learning curves and surgeon experience

Verbatim text that specifically mentioned learning curves or indicated surgeons’ experience with the interventions (for example the number of procedures performed by each surgeon every year) was extracted.

#### Assessments of bias and applicability

All included studies were assessed for validity (Risk-of-Bias (RoB)) and applicability.

##### Validity

Assessments of study validity were undertaken with the Cochrane RoB 2 tool^11^ (K.A.C., S.E.C.). Judgements of five domains were informed by the tool guidance^11^, and answers inputted into a Microsoft^®^ Excel spreadsheet with a macro to compute recommended overall RoB judgements for each trial. Verbatim text from articles was extracted and used as a narrative to support judgements. Disagreements were resolved by consensus. For this systematic review, the RoB was considered only for the primary outcome.

##### Applicability

Assessments of applicability were undertaken using the PRECIS-2 tool^12^ (K.A.C., S.E.C.). Judgements of nine domains were informed by tool guidance and scored from one (very explanatory) to five (very pragmatic) for each domain. Domains with insufficient information to enable judgement were left blank^13^. In this review, the ‘flexibility: adherence’ domain assessed the degree of fidelity by assessing reported methods used to ensure adherence to any intervention delivery protocols (for example videotaping), and if available, any unplanned crossovers and deviations from protocol. Disagreements were resolved by consensus.

### Analysis

Data were analysed and presented descriptively using averages, proportions and rates, where applicable. PRECIS scores were presented using ‘wheels’ as recommended by the tool’s authors^12^. In line with the study objectives, meta-analysis was not planned.

## Results

### Screening and included studies

**Fig. 1:**
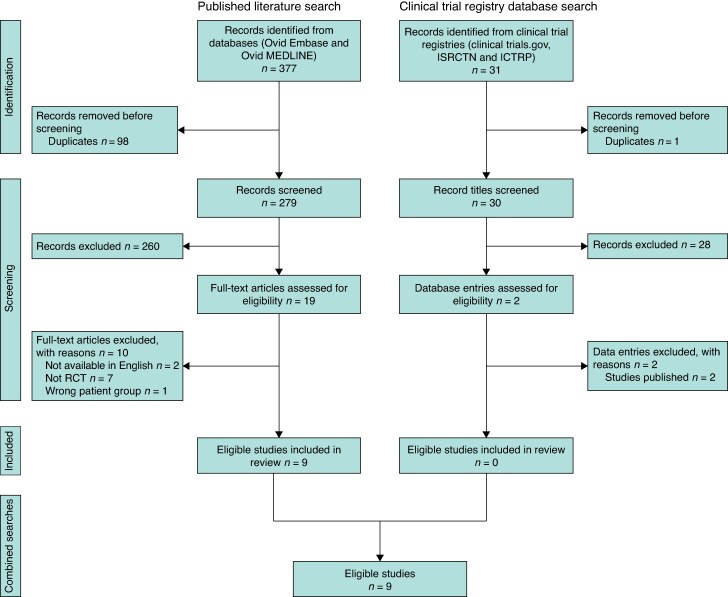
PRISMA flow chart depicting the search strategy and selection of articles from both published literature and clinical trial database searches for the review ISRCTN, International Standard Randomised Controlled Trial Number; ICTRP, International Clinical Trials Registry Platform; RCT, randomized clinical trial.

From the published literature database searches, 279 titles and abstracts were screened after the removal of duplicates. Full texts of 19 articles were reviewed and nine RCTs included^14–22^. From the clinical trial registry databases, 30 titles were screened and two entries reviewed. Neither was included as they had been published and were captured in the included texts from the literature database searches^14,15^ (*Fig. 1*).

### Key trial characteristics

*Table 1* shows the key characteristics of the nine included studies, which collectively reported outcomes in 880 participants. All trials randomized fewer than 150 patients, including three with fewer than 100 patients^16,18,20^. Only one study was conducted in multiple centres (*n* = 9)^14^. Six trials were conducted in China^15–18,21,22^, two in India^19,20^ and one in The Netherlands^14^. Two studies registered the study with a clinical trials registry^14,15^. None provided a reference or link to a trial protocol.

**Table 1:** Study characteristics

Author (year)	Sample size (centres)	Intervention (laparoscopic repair)	Comparator (open repair)	Primary outcome
Lau* (1996)^17^	103 (1)	Suture repair (suture/omentum patch) Sutureless repair (gelatin sponge secured with fibrin glue)	Suture repair (omentum patch only) Sutureless repair (gelatin sponge secured with fibrin glue)	Operative time§
Lau* (1998)^16^	22 (1)	Omental patch repair, no primary closure	Pedicled omental patch repair, no primary closure (that is Cellan-Jones)	Stress response§
Siu* (2002)^21^	130 (1)	Suture/omental patch repair	Suture/omental patch repair	Perioperative parenteral analgesic requirement
Bertleff† (2009)^14^	109 (9)	Suture repair ± omental patch	Suture repair ± omental patch	Hospital stay
Motewar‡ (2013)^19^	140 (1)	Suture/omental patch repair	Suture/omental patch repair	Complications§
Yang* (2014)^22^	106 (1)	Laparoscopic plus auxiliary small incision repair	Laparotomy	Operative time§
Shah‡ (2015)^20^	50 (1)	Suture/omental patch repair	Suture/omental patch repair	Operative time§
Ge* (2016)^15^	120 (1)	Suture only repair	Suture repair ± omental patch	Operative time#, morbidity and mortality rates
Li* (2017)^18^	100 (1)	Suture/greater omentum repair	Suture/greater omentum repair	Operative time§

*Trial conducted in China. †Trial conducted in The Netherlands. ‡Trial conducted in India. §No primary outcome stated, therefore, the first outcome reported in the abstract was assumed as the study’s primary outcome. #Primary outcome that was used to calculate required sample size.

### PICO

Most studies had broad inclusion criteria, including patients with a clinical diagnosis of PPU, with one study specifically recruiting patients with giant peptic ulcers^22^, though a definition of ‘giant’ was not provided. Three studies specified age limits/ranges (patients aged 15–70^19^, 17–69^16^ and >60^18^ years), two required evidence of air under the diaphragm on imaging^14,19^ and one excluded patients with a presentation more than 2 days after onset of symptoms^19^. Eight of the nine studies^14–16,18–22^ were two-arm trials comparing laparoscopic with open repair of PPU. One study^17^ was a four-arm trial comparing two different repair methods using both laparoscopic and open approaches.

The primary outcome of the trial was specified in three studies^14,15,21^. Five studies used operative time as the specified^15^ or assumed^17,18,20,22^ primary outcome. Across all studies, 51 outcomes were measured and reported in both the methods and results section of the article by at least one study. These represented 17 unique outcomes (analgesia, complications, duration of hospital stay, pain, quality of life, mortality rate, operative time, intraoperative bleeding, biochemical tests, time to normal diet, flatus time, antibiotic requirement, hospital cost, duration of gastrointestinal function recovery, postoperative ambulation, conversion and recovery of bowel sounds). The most outcomes measured and reported in the methods and results sections of a study was ten^18^ and the least was zero^19^ (Motewar *et al*. did not describe any outcomes to be measured in the methods^19^). All studies reported additional outcomes in the results that were not described in the methods—this was most commonly conversion rates to open surgery^14–18^. The most commonly reported secondary outcomes (by two or more studies) in either methods/results or results alone, are shown in *Table S2*, and include hospital stay^15,17,19–22^, complications^14,15,19–22^, analgesia requirement^14,15,17,20,22^ and pain^14,17,18,21,22^. The definition of outcomes described in the methods sections varied in the level of detail provided. For example, when describing complications, Bertleff *et al.* reported that ‘all complications, major and minor, were monitored’^14^ whilst Siu *et al*. stated ‘chest infections were diagnosed by radiographic evidence of pulmonary changes with or without fever >39 °C or positive cultures from sputum’^21^ (*Table S2*). Outcome measures were consistent for pain across all five studies reporting pain (visual analogue scale of 0–10)^14,17,18,21,22^. Four studies reported quality of life as the time taken to return to normal activity, but methods of measurement differed between the studies^17,18,20,21^.

### Details of intervention components and co-interventions

**Fig. 2:**
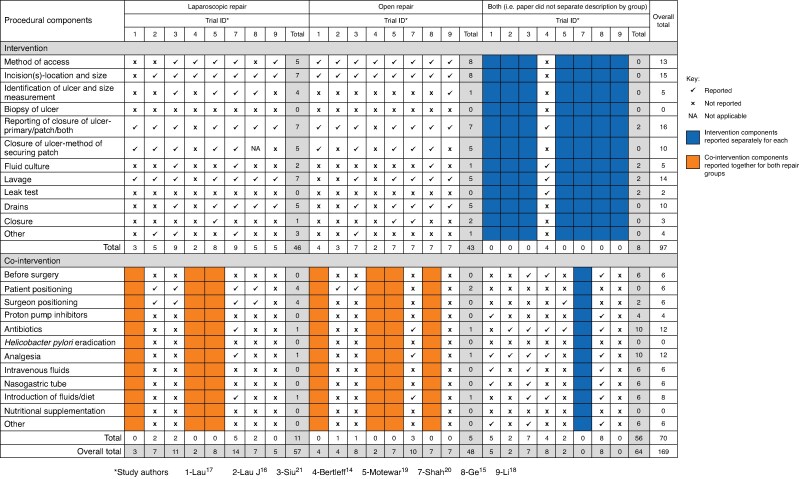
Reporting of procedural components of interventions and co-interventions for laparoscopic and open repair of perforated peptic ulcer Bold text indicates the study/studies (columns) that reported the most components and the components that were reported the most often (rows).

Methods of PPU repair were described by eight studies^14–21^ (*Table 1*). The extent to which these components were reported by each study is detailed in *Fig. 2*. Verbatim text, describing the three most reported intervention components for laparoscopic and open repair, is highlighted in *Table S3*. There were no data extracted for Yang *et al.* as it was felt that the online translation tool was not reliable for technical surgical language^22^.

#### Intervention components (laparoscopic and open repair)

The most common laparoscopic repair method combined sutures and omental patch^17–21^. Other methods included suture with or without omental patch^14^, suture only^15^, omental patch only^16^ and sutureless^17^. Open repair was performed using suture and omental patch^18–21^, suture with or without omental patch^14,15^, omental patch only^16,17^ and sutureless^17^ (*Table 1*).

Commonly reported aspects of laparoscopic repair included ‘reporting of closure of ulcer—primary, patch or both’ (*n* = 8), ‘location and size of incision’ (*n* = 7) and ‘lavage’ (*n* = 7) (*Fig. 2*). For open repair, the most reported were ‘reporting of closure of ulcer—primary, patch or both’ (*n* = 8), ‘method of access’ (*n* = 8), ‘location and size of incision’ (*n* = 8). No studies reported performing a biopsy of the ulcer (*Fig. 2*).

The descriptions of components varied in detail across studies (*Table S3*). For laparoscopic repair, of the seven studies^14–16,18–21^ that reported ‘incision—location and size’, five^15,16,19–21^ provided details about the location and size of incisions and two^14,18^ the location only. Descriptions of ‘closure of ulcer’ were variable—two studies^17,21^ provided detailed accounts including the type of needle, suture material and additional implements utilized, three reported some details such as the number of stitches^16,20^ and the needle^19^, material^16,19,20^ and knotting technique^19,20^ used whilst others^14,15,18^ provided minimal information. Lavage was either reported with^15,16,18,20,21^ or without^14,17,19^ any detail about saline and antiseptic solutions^20^. For open repair, descriptions for ‘method of access’ and ‘incision location and size’ overlapped and comprised little detail. As for the laparoscopic approach, the descriptions for ‘closure of ulcer’ were variable—one study^17^ provided an in-depth method of how closure was performed, three^18,20,21^ reported some detail about suture material^20,21^ and type of stitches^18,20,21^, one^16^ referenced the Cellan-Jones method but provided no detail and the remaining three studies^14,15,19^ reported no details of how the closure was performed.

#### Co-interventions

*Figure 2* shows that reporting of co-interventions was not as frequently reported as intervention components (72 of 192 co-interventions *versus* 99 of 192 components respectively). The most reported co-interventions were the use of antibiotics (*n* = 7) and analgesia (*n* = 7). One study^18^ did not report details of any co-interventions. Two^15,21^ studies reported 12 or more co-interventions for laparoscopic and open repair, with Ge *et al.* reporting three-quarters (18 of 24) of co-interventions^15^. Ten of the 12 suggested co-interventions were reported by at least one study. No studies reported *Helicobacter pylori* eradication or nutritional supplementation (*Fig. 2*). Nine of 12 co-interventions were reported by at least one study as being the same for both laparoscopic and open repairs.

### Details of learning curves and surgeon experience

Four studies highlighted the importance of surgeon experience in performing the laparoscopic repair^14,15,17,19^, two studies reported the grade of surgeons performing the procedures^20,21^ and three studies did not mention surgeon experience at all^16,18,22^. None of the studies required certain levels of experience for surgeons to participate in the study. *Table 2* details the verbatim text regarding surgeon experience and learning curves.

**Table 2:** Verbatim text illustrating study reporting of learning curves and/or surgeon experience

Author	Verbatim text regarding learning curve and/or surgeon experience
Lau^17^	‘This randomized study was undertaken within our normal hospital practice. We sought to avoid a comparison of one or two experienced and enthusiastic laparoscopic surgeons, with the everyday results of open repair achieved by the on-call surgeons. The study started after we had sufficient experience of the laparoscopic procedures and adequate numbers of trained staff and does not represent our initial experience in laparoscopic suture or sutureless repair. Improvements in technology and increase in laparoscopic experience may eventually change the findings of this study in future. Based on our experience, laparoscopic sutureless repair has the advantage over laparoscopic suture repair because it is technically much less demanding. The technique can be learned easily by those who have some experience with laparoscopic surgery.’
Lau^16^	Nothing reported
Siu^21^	‘Laparoscopic repair was performed by a team of four surgeons (one consultant, two senior registrars, one registrar) who were experienced in laparoscopic cholecystectomy and had undergone laboratory laparoscopic suturing training.’
Bertleff^14^	‘This (reduced conversion rate) may be partially explained by the fact that only trained and experienced laparoscopic surgeons (those performing at least 50 laparoscopic procedures a year) participated in this trial, confirming the belief that this procedure should only be done by experienced surgeons. In conclusion, the results of the LAMA trial confirm the results of other trials that laparoscopic correction of PPU is safe, feasible for the experienced laparoscopic surgeon and causes less postoperative pain.’
Motewar^19^	‘With better training in minimal access surgery now available, the time has arrived for it to take its place in the surgeon’s repertoire. Laparoscopic management of duodenal perforation required a similar average time to laparotomy with time required decreasing as the surgeons acquired more practice. With training and experience it can be performed at peripheral centres as well.’
Yang^22^	Nothing reported
Shah^20^	‘All surgeries were performed by well experienced and trained consultant-level surgeons so that all parameters can be compared uniformly.’
Ge^15^	‘In our view, this finding may be attributed largely to the proficiency of the surgical team, indicating that the surgeon’s learning curve is critical to the outcomes of patients undergoing LR surgeries.’
Li^18^	Nothing reported

LAMA, LAparoscopische MAagperforatie; PPU, perforated peptic ulcer; LR, laparoscopic repair.

Of the four studies that discussed surgeon experience^14,15,17,19^, one referred specifically to the ‘learning curve’^15^—commenting in the discussion about how the surgeon’s learning curve was critical to the outcomes of patients undergoing laparoscopic repair^15^. Bertleff *et al*.^14^ attributed the low conversion rate of laparoscopic to open repair in their study to the level of training and experience of their surgeons (50 or more laparoscopic procedures per year), and stated that the laparoscopic procedure should only be done by experienced surgeons (those performing at least 50 laparoscopic procedures a year)^14^. Motewar *et al*.^19^ reported that operative time decreased as the surgeons acquired more experience and suggested that laparoscopic repair was possible in peripheral centres with training and experience^19^. Lau^17^ reported how the study only commenced once the surgeons had sufficient experience with the laparoscopic procedure^17^—though ‘sufficient’ was not defined.

### Assessment of validity and applicability

#### Validity

**Fig. 3:**
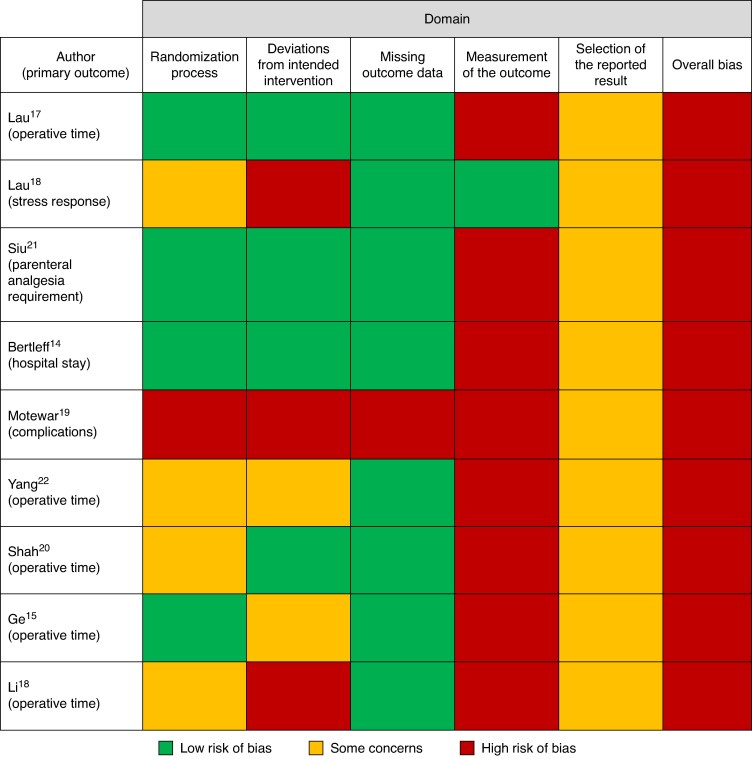
Risk-of-bias (RoB) assessments for the trial’s primary outcome, using the RoB 2 tool

All trials were judged to be at a high RoB (*Fig. 3*). The ‘measurement of the outcome’ domain was responsible for the overall high RoB judgement in eight of the nine studies^14,15,17–22^—this judgement was made as there was no information about how unblinded outcome assessors took steps to minimize introducing bias when assessing the primary outcome. No study published a protocol, meaning it was unclear if the data published in the report were the intended outcomes of interest. This meant that none of the included studies could be judged to be at low RoB.

#### Applicability

**Fig. 4:**
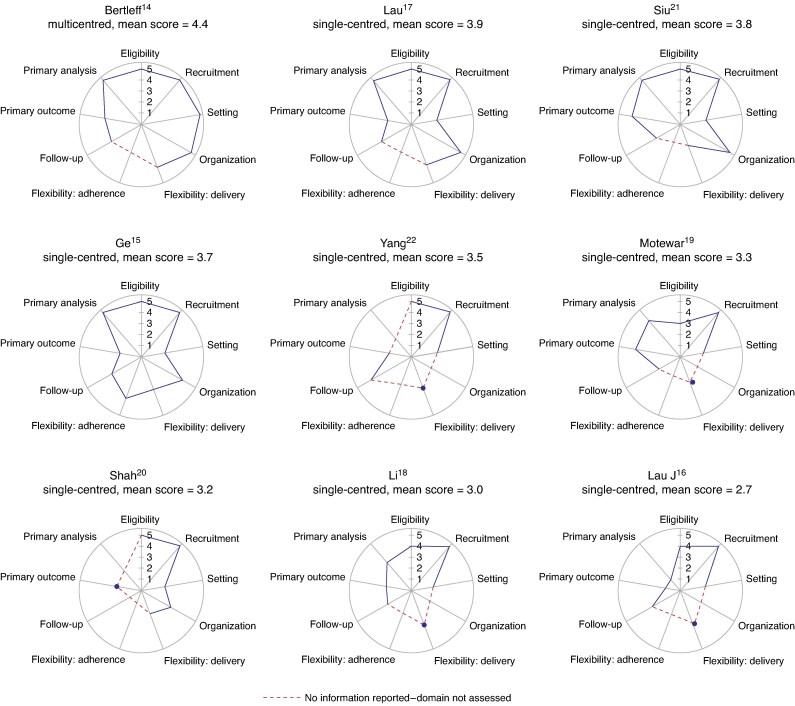
PRagmatic Explanatory Continuum Indicator Summary (PRECIS-2) scores for each domain plotted on PRECIS wheels, illustrating the pragmatism of included studies

*Figure 4* depicts the PRECIS wheels and *Fig. S1* depicts the mean PRECIS-2 scores. Inadequate reporting meant that only one study provided sufficient information to enable PRECIS assessments in all nine domains^15^; this study was the only one to report a method of monitoring the delivery of the intervention (recording of operative details). There was no information included for the ‘organization’ domain (which includes reporting of required surgeon experience) for four trials^16,18,19,22^, ‘primary analyses’ for three trials^19,20,22^ and ‘follow-up’ for one trial^20^.

The domains commonly judged to be pragmatic were ‘recruitment’ (all patients recruited from hospitals) and ‘eligibility’ (broad in the majority of studies—one study excluded patients over 70 years old and patients who had symptoms for 2 days or more^19^). Domains found to be explanatory were ‘trial setting’ (eight of the nine trials were conducted in single centres^15–22^), ‘flexibility: delivery’ (three reported detailed procedural methods suggesting little flexibility^19–21^), ‘follow-up’ (two had an intensive follow-up regimen^18,21^) and ‘primary outcome’ (seven measured primary outcomes that were not particularly patient orientated^14–18,20,22^).

Only one study was considered to have a more pragmatic than explanatory approach^14^. In addition to being multicentred, the trial reported broad eligibility criteria and brief methods suggesting flexibility in intervention delivery, and performed intention-to-treat analyses. However, a more intensive follow-up regimen than usual care and a primary outcome not wholly patient orientated (duration of hospital stay) were considered less pragmatic.

## Discussion

This review provides an in-depth examination of the existing RCTs exploring laparoscopic and open repair of PPUs. It shows that the supporting evidence for laparoscopic PPU repair is largely based on small, single-centre RCTs at high RoB, with variable patient selection, heterogenous interventions and comparators, poorly described co-interventions and outcomes of limited patient importance, compromising rigour and applicability to routine clinical practice.

Congruent with other reviews of surgical interventions^23–28^, reporting of the technical aspects of interventions and comparators was limited, even for crucial details of the procedure (such as the way the peptic ulcer was repaired) and details enabling the intervention and comparator to be distinguished from each other (that is the incision(s) and access). Moreover, there was considerable heterogeneity within the studies reporting this information, that is different details were reported for how the ulcer should be repaired. Acknowledging the age of the studies, reporting does not meet current standards such as Consolidated Standards of Reporting Trials–Non-Pharmacologic Treatments (CONSORT-NPT) or Template for Intervention Description and Replication (TIDiER)^10,29,30^, which compromises applicability and creates difficulties when attempting to replicate study findings in clinical practice.

CONSORT-NPT also expects the level of experience of participating surgeons to be reported^29^. Studies in this review either limited operating surgeons to ‘experts’, or acknowledged the existence of a learning curve, although this was not defined. Questions about learning curves and operator skillset are important when considering trial design. When intervention delivery is limited to allow only ‘expert’ operators (that is an explanatory design), the ‘best’ effects of the intervention are elicited. The alternative—allowing all surgeons with appropriate laparoscopic skills to attempt laparoscopic repair—reflects real-world implementation in a pragmatic design. A limitation of this approach is the impact on outcomes arising with inexperienced surgeons, whereas the explanatory design creates difficulties when extrapolating results beyond the ‘expert’ setting. As far as we are aware, the length of this learning curve for laparoscopic repair of PPU has not yet been established. It is important to note that studies within this review were conducted during the 1990s and early 2000s, which was relatively early in the uptake of minimally invasive surgery, and advanced laparoscopic skills may be more widely distributed in modern practice.

None of the primary outcomes reported in the included RCTs can realistically support the implementation of laparoscopic repair into routine practice. They largely focused on process metrics such as ‘operative time’ and ‘length of stay’ that are important to surgeons. Although some focused on complications, which are arguably important to patients and clinicians, patient-reported outcomes were often omitted or relegated to secondary outcomes. Inclusion of patient-focused outcomes is now considered essential for trials of this nature. The overlapping nature of the secondary outcomes list suggests that it would be feasible to establish a core outcome set^31^ for this condition. This would complement previous work in this field that has standardized the reporting of participant and disease characteristics in a consensus-defined core descriptor set^32^.

Although this review provides a comprehensive overview of all published RCTs in this area, a potential limitation of the study is the sole focus on RCTs. However, RCT is considered the ‘standard’ for research and so we sought to assess only studies at the pinnacle of the evidence pyramid. As the aim of the study is to understand previous approaches to inform future study designs, this paper was designed with a deliberately narrow scope. The study was prospectively registered on PROSPERO and uses recognized methods and tools to understand the relevance and applicability of designs.

With over 1000 patients undergoing PPU repair annually in the UK alone, many experience postoperative complications^2^. There is room to improve the treatment of these patients. Commissioners, policymakers and surgeons should be aware of the limitations of the underlying literature, and their variable applicability to practice. Adoption and implementation should be supported by regular review of outcomes and refinement where appropriate. Researchers should note the above and recognize the need for a trial that considers relevant co-interventions, is well powered and is focused on patient-important outcomes.

## Supplementary Material

zrae163_Supplementary_Data

## Data Availability

The authors confirm that the articles included in the review are fully referenced and tables and Supplementary material contain text used to underpin tool assessments.
