# Supplementary material for: Laparoscopic versus open repair of perforated peptic ulcer: systematic scoping review and in-depth evaluation of existing evidence
Source: BJS Open. 2025 Mar 6;9(2):zrae163. doi: 10.1093/bjsopen/zrae163 (PMC11882505; doi:10.1093/bjsopen/zrae163)
Supplement: zrae163_Supplementary_Data [file zrae163_supplementary_data.docx]

**Laparoscopic versus open repair of perforated peptic ulcer: a systematic scoping review and in-depth evaluation of existing evidence**

Katy A Chalmers^1^*, Matthew J Lee^2,3^*, Sian E Cousins^1^, Adam Peckham Cooper^4^, Peter O Coe^5^, Natalie S Blencowe^1,4^

**Affiliations:**

^1^ Centre for Surgical Research, University of Bristol, Bristol, UK

^2^ Institute for Applied Health Research, College of Medical and Dental Sciences, University of Birmingham, Birmingham, UK

^3^ Department of Trauma and Emergency General Surgery, University Hospitals Birmingham NHS Foundation Trust, Birmingham, UK

^4^ Leeds Institute of Emergency General Surgery, Leeds Teaching Hospital NHS Trust, Leeds, UK

^5^ Department of Upper Gastrointestinal Surgery, Leeds Teaching Hospital NHS Trust, Leeds, UK

*Katy A Chalmers and Matthew J Lee are both first authors and contributed equally

**Corresponding author:**

Katy Chalmers

Bristol Centre for Surgical Research

Canynge Hall, 39 Whatley Road

Bristol, BS8 2PS

Email: katy.chalmers@bristol.ac.uk

**ORCID ID:** https://orcid.org/0000-0003-4923-3000

**Supplementary Materials - Index**

| **Supplementary Methods** |  |
| --- | --- |
| Table S1 - search strategies | *pag. 2-3* |
| **Supplementary Results** |  |
| Table S2 - details of secondary outcomes | *pag. 4-9* |
| Table S3 - verbatim descriptions of procedural components | *pag. 10-13* |
| Figure S1 - mean domain scores for PRECIS-2 assessments | *pag. 14* |

**Supplementary Methods**

**Table S1**

**Ovid MEDLINE search strategy**

1 exp peptic ulcer/ or peptic ulcer hemorrhage/

2 (duoden* adj3 ulcer*).mp.

3 (stomach* adj3 ulcer*).mp.

4 (ulcer adj3 perforat*).mp.

5 1 or 2 or 3 or 4

6 open.tw.

7 standard.tw.

8 conventional.tw.

9 laparotomy.tw.

10 6 or 7 or 8 or 9

11 laparoscop*.mp.

12 exp laparoscopy/

13 minimally invasive surgical procedures/

14 minimal* invasive.mp.

15 single port.tw.

16 multi-port.tw.

17 11 or 12 or 13 or 14 or 15 or 16

18 controlled clinical trial.pt.

19 exp randomized controlled trial/

20 Random allocation/

21 randomi#ed.tw.

22 randomly.tw.

23 random* allocat*.tw.

24 trial.ti.

25 groups.tw.

26 18 or 19 or 20 or 21 or 22 or 23 or 24 or 25

27 5 and 10 and 17 and 26

28 limit 27 to yr="1990 -Current"

**Ovid Embase search strategy**

1 exp peptic ulcer/ or peptic ulcer hemorrhage/

2 (duoden* adj5 ulcer*).mp.

3 (stomach* adj5 ulcer*).mp.

4 (ulcer adj5 perforat*).mp.

5 1 or 2 or 3 or 4

6 open.tw.

7 standard.tw.

8 conventional.tw.

9 laparotomy.tw.

10 6 or 7 or 8 or 9

11 laparoscop*.mp.

12 exp laparoscopy/

13 minimally invasive surgical procedures/

14 minimal* invasive.mp.

15 single port.tw.

16 multi-port.tw.

17 11 or 12 or 13 or 14 or 15 or 16

18 controlled clinical trial.pt.

19 exp randomized controlled trial/

20 Random allocation/

21 randomi#ed.tw.

22 randomly.tw.

23 random* allocat*.tw.

24 trial.ti.

25 groups.tw.

26 18 or 19 or 20 or 21 or 22 or 23 or 24 or 25

27 5 and 10 and 17 and 26

28 limit 27 to yr="1990 -Current"

**Supplementary Results**

**Table S2**. Secondary outcomes reported in the methods/results section of the paper or results section only by ≥2 eligible studies and the definition of the outcome.

| **Secondary outcome^1^** | **Author** | **Definition of outcome** | |
| --- | --- | --- | --- |
|  |  | **Methods and results** | **Results** |
| Duration of hospital stay | Lau W^1^ | Number of days spent in general surgical ward |  |
|  | Lau J^2^ |  | Reported, but not defined |
|  | Siu^3^ | Number of days in the hospital after surgery, inclusive of the day of surgery |  |
|  | Bertleff^4^ |  |  |
|  | Motewar^5^ |  | Reported, but not defined |
|  | Yang^6^ | Reported, but not defined |  |
|  | Shah^7^ | Post-operative hospital stay in days |  |
|  | Ge^8^ | Total hospital stay |  |
|  | Li^9^ | Not defined, but shown as number of days |  |
| Complications | Lau W^1^ | ‘Standard' questionnaire to assess complications. Examination for surgical site infection. A wound complication was recorded if the patient reported a history of or had signs of redness around any wound or a discharge. Frequency of named complications in the Results section added up to give a morbidity score. |  |
|  | Lau J^2^ |  |  |
|  | Siu^3^ | Wound infection was defined as the presence of pus or sanguinopurulent discharge at surgical site. Chest infections were diagnosed by radiographic evidence of pulmonary changes with or without fever >39 degrees or positive cultures from sputum. Frequency of other complications reported in results. |  |
|  | Bertleff^4^ | All complications, major and minor monitored. Frequency of named complications in the Results section. |  |
|  | Motewar^5^ |  | Reported, but not defined |
|  | Yang^6^ | Incidence of a number of complications such as incision infection, inflammatory ileus, cracking of incision, intestinal space abscess, pelvic abscess and lung infections |  |
|  | Shah^7^ | Number of patients experiencing leak, wound infection or pelvic abscess |  |
|  | Ge^8^ |  |  |
|  | Li^9^ | Incidence of post-operative complications calculated. Frequency of named complications in the Results section. |  |
| Analgesia requirement | Lau W^1^ | Number of doses of pethidine in previous 24 hours |  |
|  | Lau J^2^ |  | Reported, but not defined |
|  | Siu^3^ |  |  |
|  | Bertleff^4^ | Days opiates used |  |
|  | Motewar^5^ |  |  |
|  | Yang^6^ | Reported, not defined, but shown as utilization rate |  |
|  | Shah^7^ | Analgesia requirement in days |  |
|  | Ge^8^ | Post-op PCA consumption |  |
|  | Li^9^ |  |  |
| Pain | Lau W^1^ | VAS (10cm line without graduations) for pain in first 24 hours of the operation |  |
|  | Lau J^2^ |  |  |
|  | Siu^3^ | VAS (10cm line without graduations) on days 1 and 5 |  |
|  | Bertleff^4^ | VAS (0-10) on days 1, 3, 7 and 28 ranging from 0 (no pian) to 10 (severe pain) |  |
|  | Motewar^5^ |  |  |
|  | Yang^6^ | VAS score at 1, 3 and 7 days after surgery |  |
|  | Shah^7^ |  |  |
|  | Ge^8^ |  |  |
|  | Li^9^ | VAS (0 to 10) 0 (painless) to 10 (severe pain) before surgery and 1 and 3 days after surgery |  |
| Quality of life | Lau W^1^ | ‘Standard' questionnaire to assess return to normal activity and return to work. Return to normal activity is defined as return to normal daily activity without assistance. |  |
|  | Lau J^2^ |  |  |
|  | Siu^3^ | Time to return to normal activity in days (patients kept a diary of the date of resumption of full daily activities and work |  |
|  | Bertleff^4^ |  |  |
|  | Motewar^5^ |  |  |
|  | Yang^6^ |  |  |
|  | Shah^7^ | Return to normal physical activities in days |  |
|  | Ge^8^ |  |  |
|  | Li^9^ | Quality of life questionnaires covers 45 scores ((1-5 for each of 8 categories) (spirit, fatigue, appetite, pain, sleep, attitude towards treatment, side effects of treatment and daily life)) |  |
| Operative time | Lau W^1^ |  |  |
|  | Lau J^2^ |  | Reported, but not defined |
|  | Siu^3^ | Time from the first incision to the placement of the last suture |  |
|  | Bertleff^4^ |  | Reported, but not defined |
|  | Motewar^5^ |  | Reported, but not defined |
|  | Yang^6^ |  |  |
|  | Shah^7^ |  |  |
|  | Ge^8^ |  |  |
|  | Li^9^ |  |  |
| Intraoperative bleeding | Lau W^1^ |  |  |
|  | Lau J^2^ |  |  |
|  | Siu^3^ |  |  |
|  | Bertleff^4^ |  | Not defined, but shown as volume in ml |
|  | Motewar^5^ |  |  |
|  | Yang^6^ | Not defined, but shown as volume in ml |  |
|  | Shah^7^ |  |  |
|  | Ge^8^ |  |  |
|  | Li^9^ | Not defined, but shown as volume in ml |  |
| Mortality | Lau W^1^ |  | Reported, but not defined |
|  | Lau J^2^ |  |  |
|  | Siu^3^ | Reported, but not defined |  |
|  | Bertleff^4^ |  |  |
|  | Motewar^5^ |  | Reported, but not defined |
|  | Yang^6^ |  |  |
|  | Shah^7^ |  |  |
|  | Ge^8^ |  |  |
|  | Li^9^ |  |  |
| Biochemical tests | Lau W^1^ |  |  |
|  | Lau J^2^ | Assays for plasma endotoxin and bacteria count |  |
|  | Siu^3^ |  |  |
|  | Bertleff^4^ |  |  |
|  | Motewar^5^ |  |  |
|  | Yang^6^ |  |  |
|  | Shah^7^ |  |  |
|  | Ge^8^ |  |  |
|  | Li^9^ | Assays for hs-CRP, IL-6 and TNF-α |  |
| Flatus time | Lau W^1^ |  |  |
|  | Lau J^2^ |  |  |
|  | Siu^3^ |  |  |
|  | Bertleff^4^ |  |  |
|  | Motewar^5^ |  |  |
|  | Yang^6^ | Not defined, but shown as time in hours |  |
|  | Shah^7^ |  |  |
|  | Ge^8^ |  |  |
|  | Li^9^ | Not defined, but shown as time in hours |  |
| Post-operative ambulation | Lau W^1^ |  |  |
|  | Lau J^2^ |  |  |
|  | Siu^3^ |  |  |
|  | Bertleff^4^ |  |  |
|  | Motewar^5^ |  |  |
|  | Yang^6^ | Not defined, but shown as time in hours |  |
|  | Shah^7^ |  |  |
|  | Ge^8^ |  |  |
|  | Li^9^ | Not defined, but shown as time in hours |  |

**Abbreviations:** hs-CRP – high-sensitivity C-reactive protein, IL-6 – interleukin-6, PCA – patient-controlled analgesia, TNF-α – tumour necrosis factor-α, VAS – visual analogue scale

^1^ Studies reporting these outcomes as primary outcomes, or assumed primary outcomes, are not included in the table.

**Table S3.** Descriptions of the most reported components

***Laparoscopic repair***

| **Component** | **Author** | **Verbatim description of component** |
| --- | --- | --- |
| **Incision – location and size** | Lau W^1^ | Nothing reported |
|  | Lau J^2^ | “Hassan’s blunt cannula was placed in the umbilicus with an open technique… An 11-mm trocar and a 5-mm trocar were inserted in the right and left flanks, respectively. A 5-mm trocar was inserted to the right of falciform ligament in the epigastrium” |
|  | Siu^3^ | “The open method was used for insertion of the initial 10-mm umbilical port. A 30° laparoscope was then introduced. Two additional working ports were inserted at the level of the transpyloric plane at the midclavicular line on both sides. A 10-mm cannula was inserted in the left subcostal region to facilitate the insertion of sutures” |
|  | Bertleff^4^ | “Trocars were placed at the umbilicus (video scope) and on the left and right midclavicular line above the level of the umbilicus (instruments). If necessary, a fourth trocar was placed in the subxiphoid space for lavage or retraction of the liver” |
|  | Motewar^5^ | “…insertion of 4 ports: 10 mm umbilical, 10 mm epigastric, 5 mm medial to left mid-clavicular line and 5 mm in right mid-clavicular line.” |
|  | Shah^7^ | “Standard four port technique: 10mm umbilical, three 5-mm ports were inserted—two in the midclavicular line on the left side and one on the right side.” |
|  | Ge^8^ | “…established an infraumbilical stab incision in the pneumoperitoneum using a Veress needle. The laparoscope was then introduced through a 10-mm trocar. Two additional working ports were inserted at the level of the transpyloric plane at the midclavicular line on both sides, and a 10-mm cannula was inserted in the left subcostal region, to facilitate the insertion of sutures” |
|  | Li^9^ | “a 1 cm arc incision was made below the umbilicus to establish pneumoperitoneum where a laparoscope was inserted through the umbilicus, followed by puncture and insertion of the trocars into the sites apart below the left costal margin at the midclavicular line and below the right costal margin at the anterior axillary line.” |
| **Closure of ulcer – primary, patch or both** | Lau W^1^ | “Suturing was done with a needle holder. The needle was passed through the duodenum near the perforation and through a mobilized patch of omentum. An extracorporeal Roeder knot'^5^ was tied in the suture and passed down to fix the patch over the perforation. Additional sutures were passed, as required, to surround the perforation. This was followed by peritoneal lavage with normal saline // The technique of laparoscopic repair with gelatin sponge and fibrin glue has been reported.'^4^ A piece of gelatin sponge (Spongostan; Ferrosan, Soeborg, Denmark) 20 X 15 X 10 mm thick sheet was rolled into a one. This plug was grasped with a forceps and backloaded into a 10-mm reducing sheath for insertion into the abdominal cavity. The plug was placed into the perforation so that the base of the cone protruded onto the serosal surface. A prewarmed 2-mL volume of two-component fibrin sealant (Tisseel; Immuno, Vienna, Austria) was injected slowly via a double lumen catheter around the plug to secure it” |
|  | Lau J^2^ | “Two to three interrupted 2/0 polydiaxanone sutures were passed over the perforation. A piece of omentum was held in place and tied over the perforation.” |
|  | Siu^3^ | “We have reported our single-stitch laparoscopic repair method previously.^26^ A Szabo-Berci “parrot jaws” needle driver was used for suturing. The needle and suture were introduced through the 10-mm operating port on the left. Liver retraction was maintained by blunt dissection forceps placed in the right subcostal port. A single stitch (3-0 polygalactin half-circle round-bodied needle) was applied with a good bite of full-thickness healthy tissue taken longitudinally across the middle of the perforations. The ulcer edges were approximated by intracorporeal knotting, with two identical half knots forming a square knot followed by a third and opposite half knot.” |
|  | Bertleff^4^ | “Closure of PPU was to be achieved by sutures alone or in combination with an omental patch.” |
|  | Motewar^5^ | “…2.0 silk using a round body needle and intra-corporeal knotting and a live omental patch was kept.” |
|  | Shah^7^ | “The perforation was closed using two to three interrupted 3-0 Vicryl sutures tied over the omentum patch using intracorporeal knot-tying technique.” |
|  | Ge^8^ | “…perforation was closed with simple interrupted sutures without using pedicled omentoplasty.” |
|  | Li^9^ | “The duodenal perforations were treated using repair of perforation while the gastric perforation was repaired with the use of interrupted sutures; then the sites of perforation were covered by the greater omentum.” |
| **Lavage** | Lau W^1^ | “Peritoneal lavage was performed before completion of the operation.” |
|  | Lau J^2^ | “Peritoneal lavage was performed with at least 4L warm normal saline or until aspirate returned clear.” |
|  | Siu^3^ | “Thorough peritoneal lavage was performed with pressurized warm normal saline. all the purulent exudates and irrigation fluid were aspirated before closure“ |
|  | Bertleff^4^ | “…after which the peritoneal cavity was lavaged.” |
|  | Motewar^5^ | “All intra-abdominal cavities were thoroughly irrigated, and suction was done” |
|  | Shah^7^ | “…thorough peritoneal lavage was done with warm normal saline and Betadine” |
|  | Ge^8^ | “Thorough peritoneal lavage was then accomplished by systematic warm saline infusion, and aspiration of the peritoneal fluid.” |
|  | Li^9^ | “The peritoneal and the pelvic cavities were flushed by normal saline” |

***Open repair***

| **Component** | **Author** | **Verbatim description of component** |
| --- | --- | --- |
| **Method of access** | Lau W^1^ | “Through an upper midline incision...“ |
|  | Lau J^2^ | “Upper midline incisions were made” |
|  | Siu^3^ | “All open repairs were performed according to standard techniques described in surgical textbooks“ |
|  | Bertleff^4^ | “The open surgical procedure was performed through an upper abdominal midline incision” |
|  | Motewar^5^ | “laparotomy” |
|  | Shah^7^ | “A standard upper paramedian laparotomy” |
|  | Ge^8^ | “The operative procedure for OR was performed through an upper abdominal midline incision.” |
|  | Li^9^ | “Laparotomy - Under general anesthesia, an incision was cut through the right upper abdominal of the patient“ |
| **Incision – location and size** | Lau W^1^ | “Through an upper midline incision.“ |
|  | Lau J^2^ | “Upper midline incisions were made” |
|  | Siu^3^ | “We used an upper midline incision” |
|  | Bertleff^4^ | “The open surgical procedure was performed through an upper abdominal midline incision” |
|  | Motewar^5^ | “standard exploratory laparotomy” |
|  | Shah^7^ | “A standard upper paramedian laparotomy” |
|  | Ge^8^ | “The operative procedure for OR was performed through an upper abdominal midline incision.” |
|  | Li^9^ | “an incision was cut through the right upper abdominal of the patient, to gain access into the peritoneal cavity by separating layers of skin and muscle tissues.” |
| **Closure of ulcer – primary, patch or both** | Lau W^1^ | “Through an upper midline incision, a wad of omentum was drawn under an arch of full-thickness absorbable sutures placed on either side of the perforation, and then the sutures were tied// Through an upper midline incision, the repair was done with gelatin sponge and fibrin glue as in group 2. (A piece of gelatin sponge (Spongostan; Ferrosan, Soeborg, Denmark) 20 X 15 X 10 mm thick sheet was rolled into a one. This plug was grasped with a forceps and backloaded into a 10-mm reducing sheath for insertion into the abdominal cavity. The plug was placed into the perforation so that the base of the cone protruded onto the serosal surface. A prewarmed 2-mL volume of two-component fibrin sealant (Tisseel; Immuno, Vienna, Austria) was injected slowly via a double lumen catheter around the plug to secure it”) |
|  | Lau J^2^ | “Perforations were repaired with the Cellan-Jones method.” |
|  | Siu^3^ | “a healthy piece of omentum was drawn under an arch of full-thickness polygalactin sutures placed on either side of the perforation, and the sutures were tied“ |
|  | Bertleff^4^ | “Closure of PPU was to be achieved by sutures alone or in combination with an omental patch.” |
|  | Motewar^5^ | “…primary closure of duodenal perforation and also application of a live omental patch were done.” |
|  | Shah^7^ | “…closed in 3-0 Vicryl intermittent stitches and omental patch was kept after it.” |
|  | Ge^8^ | “Closure of PPU was achieved by sutures alone or in combination with an omental patch.” |
|  | Li^9^ | “The perforations were sutured using interrupted sutures and finally covered by the greater omentum.“ |

**Figure S1.** Mean domain scores for PRECIS-2^10^ (PRagmatic Explanatory Continuum Indicator Summary) assessments for each trial.

|  | **PRECIS-2 domains** | | | | | | | | | |
| --- | --- | --- | --- | --- | --- | --- | --- | --- | --- | --- |
| **First author** | **Eligibility** | **Recruitment** | **Setting** | **Organisation** | **Flexibility: delivery** | **Flexibility: adherence** | **Follow-up** | **Primary outcome** | **Primary analyses** | **Mean score (trial)** |
| Lau W^1^ | 5 | 5 | 2 | 5 | 4 | NR | 3 | 2 | 5 | 3.9 |
| Lau J^2^ | 4 | 5 | 2 | NR | 3 | NR | 3 | 1 | 1 | 2.7 |
| Siu^3^ | 5 | 5 | 2 | 5 | 2 | NR | 2 | 4 | 5 | 3.8 |
| Bertleff^4^ | 5 | 5 | 5 | 5 | 4 | NR | 3 | 3 | 5 | 4.4 |
| Motewar^5^ | 3 | 5 | 2 | NR | 2 | NR | 4 | 4 | NR | 3.3 |
| Yang^6^ | 5 | 5 | 2 | NR | 3 | NR | 4 | 2 | NR | 3.5 |
| Shah^7^ | 5 | 5 | 2 | 3 | 2 | NR | NR | 2 | NR | 3.2 |
| Ge^8^ | 5 | 5 | 2 | 4 | 3 | 4 | 3 | 2 | 5 | 3.7 |
| Li^9^ | 4 | 5 | 2 | NR | 3 | NR | 2 | 2 | 3 | 3.0 |
| **Mean score (domain)** | 4.6 | 5.0 | 2.3 | 4.4 | 2.9 | 4.0 | 3.0 | 2.4 | 4.0 |  |

Pragmatic

Equally pragmatic and explanatory

Explanatory

**References**

1. Lau WY, Leung KL, Kwong KH, Davey IC, Robertson C, Dawson JJW, et al. A randomized study comparing laparoscopic versus open repair of perforated peptic ulcer using suture or sutureless technique. *Annals of Surgery* 1996;**224**(2): 131-138.

2. Lau JYW, Lo Mphil SY, Ng EKW, Lee DWH, Lam YH, Chung SCS. A randomized comparison of acute phase response and endotoxemia in patients with perforated peptic ulcers receiving laparoscopic or open patch repair. *American Journal of Surgery* 1998;**175**(4): 325-327.

3. Siu WT, Leong HT, Law BKB, Chau CH, Li ACN, Fung KH, et al. Laparoscopic repair for perforated peptic ulcer: A randomized controlled trial. *Annals of Surgery* 2002;**235**(3): 313-319.

4. Bertleff MJOE, Halm JA, Bemelman WA, Van Der Ham AC, Van Der Harst E, Oei HI, et al. Randomized clinical trial of laparoscopic versus open repair of the perforated peptic ulcer: The LAMA trial. *World Journal of Surgery* 2009;**33**(7): 1368-1373.

5. Motewar A, Tilak M, Patil DS, Bhamare N, Bhople L. Laparoscopic versus open management of duodenal perforation: A comparative study at a District General Hospital. *Przeglad Gastroenterologiczny* 2013;**8**(5): 315-319.

6. Yang XZ, Chen LY. Laparoscopic-assisted surgery for perforated peptic ulcer: Analysis of 53 cases. *World Chinese Journal of Digestology* 2014;**22**(14): 2055-2058.

7. Shah FH, Mehta SG, Gandhi MD, Saraj. Laparoscopic Peptic Ulcer Perforation Closure: the Preferred Choice. *The Indian journal of surgery* 2015;**77**(Suppl 2): 403-406.

8. Ge B, Wu M, Chen Q, Lin R, Liu L, Huang Q. A prospective randomized controlled trial of laparoscopic repair versus open repair for perforated peptic ulcers. *Surgery (United States)* 2016;**159**(2): 451-458.

9. Li J, Shang X, Zheng X. Laparoscopy versus laparotomy for upper gastrointestinal perforation in elderly patients. *International Journal of Clinical and Experimental Medicine* 2017;**10**(8): 11991-11997.

10. Loudon K, Treweek S, Sullivan F, Donnan P, Thorpe KE, Zwarenstein M. The PRECIS-2 tool: designing trials that are fit for purpose. *BMJ* 2015;**350**: h2147.
